# Acute-to-chronic glycemic ratio as an outcome predictor in ischemic stroke in patients with and without diabetes mellitus

**DOI:** 10.1186/s12933-024-02260-9

**Published:** 2024-06-18

**Authors:** Elisenda Climent, Ana Rodriguez-Campello, Joan Jiménez-Balado, Mercè Fernández-Miró, Jordi Jiménez-Conde, Gemma Llauradó, Ángel Ois, Juana A. Flores, Elisa Cuadrado-Godia, Eva Giralt Steinhauer, Juan J. Chillarón, Neurovascular Research Group (NEUVAS), Isabel Fernandez Perez, Adrià Macías Gomez, Antoni Suarez Perez, Daniel Guisado Alonso, Marta Vallverdú Prats

**Affiliations:** 1 Endocrinology and Nutrition Department, Hospital del Mar, Barcelona, Spain; 2 IMIM (Hospital del Mar Medical Research Institute), Barcelona, Spain; 3 Department of Medicine (MELIS), Universitat Pompeu Fabra, Barcelona, Spain; 4 Neurology Department, Hospital del Mar, Barcelona, Spain; 5 Neurovascular Research Group (NEUVAS), Neurology Department, Hospital del Mar, Barcelona, Spain; 6 Centro de Investigación Biomédica en Red de Diabetes y Enfermedades Metabólicas Asociadas (CIBERDEM), Instituto de Salud Carlos III, Madrid, Spain

**Keywords:** Acute-to-chronic glycemic ratio, Diabetes, Hyperglycemia, Ischemic stroke, Mortality, outcome

## Abstract

**Objective:**

Elevated plasma glucose levels are common in patients suffering acute ischemic stroke (AIS), and acute hyperglycemia has been defined as an independent determinant of adverse outcomes. The impact of acute-to-chronic glycemic ratio (ACR) has been analyzed in other diseases, but its impact on AIS prognosis remains unclear. The main aim of this study was to assess whether the ACR was associated with a 3-month poor prognosis in patients with AIS.

**Research, design and methods:**

Retrospective analysis of patients admitted for AIS in Hospital del Mar, Barcelona. To estimate the chronic glucose levels (CGL) we used the formula eCGL= [28.7xHbA1c (%)]-46.7. The ACR (glycemic at admission / eCGL) was calculated for all subjects. Tertile 1 was defined as: 0.28–0.92, tertile 2: 0.92–1.13 and tertile 3: > 1.13. Poor prognosis at 3 months after stroke was defined as mRS score 3–6.

**Results:**

2.774 subjects with AIS diagnosis were included. Age, presence of diabetes, previous disability (mRS), initial severity (NIHSS) and revascularization therapy were associated with poor prognosis (p values < 0.05). For each 0.1 increase in ACR, there was a 7% increase in the risk of presenting a poor outcome. The 3rd ACR tertile was independently associated with a poor prognosis and mortality. In the ROC curves, adding the ACR variable to the classical clinical model did not increase the prediction of AIS prognosis (0.786 vs. 0.781).

**Conclusions:**

ACR was positively associated with a poor prognosis and mortality at 3-months follow-up after AIS. Subjects included in the 3rd ACR tertile presented a higher risk of poor prognosis and mortality. Baseline glucose or ACR did not add predictive value in comparison to only using classical clinical variables.

**Supplementary Information:**

The online version contains supplementary material available at 10.1186/s12933-024-02260-9.

## Introduction

The inflammatory response secondary to the presence of an acute disease can lead to an increase in glucose levels, known as stress hyperglycemia [1]. Previous research confirmed that this glucose disturbance could play a role in the clinical prognosis of certain diseases. For instance, stress hyperglycemia predicts mortality in subjects with established cardiovascular disease, both in patients with and without diabetes [2, 3].

Acute hyperglycemia is a frequent condition observed in patients with an acute ischemic stroke (AIS) [4]. However, contrary to other cardiovascular diseases, the exact role of stress hyperglycemia in subjects after stroke diagnosis has not been fully characterized [5]. This may be explained, at least in part, since previous publications are scarce and these have mostly included non-diabetic subjects [6] or have only evaluated fasting blood glucose (FBG) [7].

Regarding stress hyperglycemia assessment, different ratios and indexes have been recently described to evaluate the balance between chronic and acute glucose control, and assess the impact of stress hyperglycemia on clinical prognosis. In this regard, the acute-to-chronic glycemic ratio (ACR) is calculated as the glucose at admission divided by chronic glucose levels. This ratio has shown a good correlation with illness severity in patients with acute myocardial infarction or infection [8, 9]. Moreover, in our population, ACR was found to be a predictive factor of poor functional outcomes both in subjects with COVID-19 infection [10] and heart failure [11] diagnoses. As to neurological patients, ACR is known to be linked to stroke severity in patients with diabetes [12], although no previous studies have evaluated the role of ACR as a stroke-prognostic factor in subjects with and without diabetes.

The main aim of the present study was to assess the association between ACR and 3-month poor prognosis in patients with AIS, with or without previous diabetes diagnosis. As secondary aims, the presence of other factors independently associated with mortality and poor prognosis at 3 months, and the differences between subjects with AIS diagnosis according to the 3 distinct ACR tertiles were also evaluated. Finally, we assessed whether adding glucose at admission or ACR added extra value to a predictive model based on classical clinical variables.

## Research, design and methods

### Study design and participants

The present study evaluates patients admitted for ischemic stroke, based on data from a prospective cohort. We analyzed data from Basicmar, a prospective stroke registry at Hospital del Mar, a tertiary stroke center with a catchment area of 330,000 inhabitants covering three of the 10 districts of Barcelona. Basicmar is a consecutive database that collects information on all patients presenting with a diagnosis of stroke and transient ischemic attacks. The diagnosis of stroke was confirmed by a neurologist. Stroke severity was defined by the National Institute of Health Stroke Scale (NIHSS) at admission. Radiological studies were performed using computed tomography (CT) or magnetic resonance imaging (MRI) to exclude other causes.

The database includes demographic data, sex, age, medication, and cardiovascular risk factors. Information on cardiovascular risk factors was obtained from the patient, family, or medical history. A vascular neurologist classified ischemic stroke as atherothrombotic, cardioembolic, lacunar, unusual, or undetermined according to the etiological criteria of the Trial of Org 10,172 in Acute Stroke Treatment (TOAST) [13]. Revascularization treatment was defined as intravenous thrombolysis alone (IVT) or mechanical thrombectomy (MT) with or without thrombolysis.

Data collection for the study followed local research ethics guidelines. The identity of individual patients was completely anonymized. The study was approved by the local ethics committee (CEIC-Parc de Salut Mar, Barcelona, Spain). All participants or their approved proxy provided written informed consent for participation. The study was conducted according to the principles expressed in the Declaration of Helsinki and the applicable national legislation.

Data on disability was obtained as pre-stroke functional status during the initial medical visit at admission either directly with the patient or relatives and at 3-month follow-up medical visit or by a telephone call from the medical team using the modified Rankin Scale (mRS). Data on mortality (mRS 6) were obtained from medical records, telephone contact with the primary care physician, or family members. All subjects were independent and presented a mRS score 0–2 before stroke diagnosis.

### Definitions

The following definitions were used to classify patients: hypertension: previous medical history, use of antihypertensive medication, or evidence of hypertension in two different measurements (systolic blood pressure > 140 mmHg and diastolic blood pressure > 90 mmHg); diabetes mellitus: previous medical history, use of hypoglycemic medication, glycosylated hemoglobin (HbA1c) > 6.5%, or medical diagnosis during follow-up; dyslipidemia: previous medical history, use of medication, serum total cholesterol > 220 mg/dL, or triglycerides > 150 mg/dL; coronary artery disease: previous medical history of myocardial infarction or angina pectoris; smoking: active smoking.

Average chronic glucose levels were estimated by HbA1c, expressed as a percentage value, according to the following validated formula​ [3]:

$$\text{Estimated chronic glucose levels (mg/dL)} = (28.7 \times \text{HbA1c } \%) - 46.7$$

The ACR was calculated in all patients as glucose at admission (mg/dL) / estimated chronic glucose levels (mg/dL). Patients were stratified into 3 groups according to ACR tertiles (tertile 1: 0.28–0.92, tertile 2: 0.92–1.13 and tertile 3: > 1.13).

To convert glucose mg/dL to mmol/L (SI), a conversion factor of 0.0555 was used.

### Outcome measures

Stroke prognosis was defined as good prognosis (mRS 0–2) or poor prognosis (mRS 3–6). Mortality was defined as mRS 6 during the 3 months after AIS diagnosis.

The primary endpoint was to assess the relationship between ACR and 3-months poor prognosis and mortality after AIS diagnosis.

Secondary endpoints included: evaluation of other possible factors independently associated with mortality and poor prognosis at 3 months after AIS diagnosis, assess the differences between subjects according to the 3 distinct ACR tertiles and evaluate the relevance of adding baseline glucose or ACR to a classical model of stroke prognosis prediction.

### Statistical analysis

Data were presented as median (interquartile range) or frequency (percentage) for continuous and categorical variables, respectively. As ACR presented a right skewed distribution, we applied the log transformation to normalize it (log-ACR). Additionally, ACR was categorized according to the tertiles distribution. We used bivariate analyses (χ^2^, U Man-Witney or Kruskal-Wallis tests) to determine which factors were associated with categorical ACR and poor stroke outcome. Cohen’s d statistic was used in the univariate analyses to measure the effect size of a difference in log-ACR between patients with poor and good outcomes. To study whether log- and categorical ACR were independently associated with poor prognosis and mortality at 3 months, we used binary logistic regression models. These models were adjusted for those variables that were significantly associated with stroke outcome or mortality in the univariate analyses or which are known to be involved in stroke recovery. Therefore, we adjusted for age, sex, previous mRS, baseline NIHSS, stroke treatment and diabetes. Log- and categorical ACR were entered as predictors of interest in separate models for each dependent variable (stroke prognosis and mortality). Additionally, for each model we tested whether a significant interaction between ACR and diabetes existed. All models were interrogated for statistical assumptions: linear association between continuous predictors and log-odds and absence of concerning collinearity and influential cases.

Our next objective was to assess whether we obtained an improvement of predictions by the inclusion of ACR to a clinical model predicting the risk of poor prognosis or mortality. First, our sample was split into a train (75%) and test (25%) sets. For building the clinical models, candidate variables were those clinical factors that might be involved in stroke recovery and which are available within the first 24 h after stroke onset: age, sex, hypertension, diabetes, dyslipidemia, previous ischemic cardiopathy, atrial fibrillation, systolic blood pressure, initial NIHSS, previous mRS and stroke treatment. We then tested in the training set all the possible combinations of variables and selected the model that minimized the Aikake Index Criterion (AIC). This model was used as our baseline model. We subsequently built two additional models for each endpoint: [1] adding log-ACR to the baseline clinical model; [2] adding baseline glucose. Therefore, we could compare the advantage of adding ACR to a clinical model as compared to the addition of acute baseline glucose. We finally compared the performance of these models in the test dataset by observing the area under the ROC curve as well as other metrics of interest (accuracy, sensitivity, specificity and F1-score). Differences in ROC curves between models were tested via DeLong’s tests.

## Results

### Global baseline characteristics

A total of 2.774 subjects admitted to hospital with AIS diagnosis were included in the final study. The percentage of males was 55.8%, with a median age of 74 (64–82) years. Average HbA1c value in the sample was 5.8% (5.4–6.6%), plasma glucose at admission was 121 mg/dL (103-158 mg/dL) and median baseline ACR was 1.02 (0.88-1-21). In patients without diabetes, the mean glucose values at admission were 112 (99–130) mg/dL and HbA1c 5–5% (5.2–5.8%), while in diabetic patients, they were 164 (127-221.5) mg/dL and 7.2% (6.4–8.3%), respectively.

Almost 75% and 35% of the subjects had previous hypertension and diabetes diagnosis, respectively. Focusing on those subjects with previous diabetes diagnosis, 15.3% and 65.5% had hypertension and dyslipidemia, respectively, and 54.2% presented HbA1c levels > 7%. Regarding stroke diagnosis, 32% of patients had a cardioembolic origin, with a median baseline NIHSS of 4 [2–9] (Table 1).

**Table 1:** Baseline characteristics of the total cohort (*N* = 2774)

Baseline characteristics	Global	ACR Tertile 1 (0.28–0.92)	ACR Tertile 2 (0.92–1.13)	ACR Tertile 3 (> 1.13)	*p* value
N	2774	916	912	942	
Sex, male	1547 (55.8)	532 (58.1)	507 (55.7)	506 (53.7)	0.157
Age, years	74.0 (64.0–82.0)	73.0 (62.0–81.0)	75.0 (64.0–82.0)	75.0 (65.0–82.0)	**0.028**
Hypertension	2062 (74.4)	672 (73.4)	665 (73.0)	723 (76.8)	0.123
Dyslipidemia	1407 (50.9)	507 (55.7)	420 (46.1)	479 (51.1)	**< 0.001**
Diabetes	959 (34.6)	340 (37.1)	239 (26.2)	376 (39.9)	**< 0.001**
Coronary heart disease	399 (14.5)	146 (16.0)	111 (12.2)	142 (15.1)	0.053
Atrial fibrillation	844 (30.4)	239 (26.1)	290 (31.8)	312 (33.1)	**0.002**
BMI (kg/m^2^)	26.8 (24.2–29.8)	27.0 (24.5–29.9)	26.6 (23.9–29.8)	27.0 (24.2–29.7)	0.440
Systolic blood pressure (mmHg)	154 (137–175)	151 (135–173)	153 (137–175)	155 (139–178)	**0.042**
Diastolic blood pressure (mmHg)	80.0 (70.0–91.0)	80.0 (70.0–90.0)	80.0 (71.0–91.0)	80.0 (70.0–91.0)	0.291
rtPA treatment	531 (19.5)	155 (17.3)	176 (19.7)	200 (21.5)	0.070
Endovascular treatment	663 (24.2)	187 (20.7)	221 (24.6)	255 (27.4)	**0.003**
Baseline NIHSS	4.00 (2.00–9.00)	3.00 (2.00–7.00)	4.00 (2.00–9.00)	5.00 (2.00–12.0)	**< 0.001**
*TOAST*					**< 0.001**
Atherotrombotic	425 (15.3)	156 (17.0)	129 (14.1)	139 (14.8)	
Cardioembolic	887 (32.0)	239 (26.1)	300 (32.9)	345 (36.6)	
Lacunar	631 (22.7)	234 (25.5)	219 (24.0)	178 (18.9)	
Undetermined	738 (26.6)	259 (28.3)	235 (25.8)	244 (25.9)	
Unusual	93 (3.35)	28 (3.06)	29 (3.18)	36 (3.82)	
*Baseline mRS*					0.756
0	2012 (72.5)	660 (72.1)	667 (73.1)	681 (72.3)	
1	392 (14.1)	132 (14.4)	133 (14.6)	127 (13.5)	
2	370 (13.3)	124 (13.5)	112 (12.3)	134 (14.2)	
Glucose (mg/dL)	121 (103–158)	101 (91.0-117)	116 (106–134)	162 (133–210)	**< 0.001**
HbA1c (%)	5.80 (5.40–6.60)	6.00 (5.60–6.90)	5.70 (5.30–6.20)	5.70 (5.20–6.80)	**< 0.001**
HbA1c (mmol/mol)	40 (36–49)	42 (38–52)	39 (34–44)	39 (33–51)	**< 0.001**
ACR	1.02 (0.88–1.21)	0.82 (0.74–0.88)	1.02 (0.97–1.06)	1.33 (1.20–1.49)	**< 0.001**
*mRS during hospitalization*					**< 0.001**
0	644 (23.2)	239 (26.1)	211 (23.1)	193 (20.5)	
1	590 (21.3)	211 (23.0)	193 (21.2)	186 (19.7)	
2	594 (21.4)	225 (24.6)	199 (21.8)	170 (18.0)	
3	403 (14.5)	115 (12.6)	136 (14.9)	152 (16.1)	
4	261 (9.4)	73 (7.97)	87 (9.54)	100 (10.6)	
5	46 (1.6)	7 (0.76)	13 (1.43)	26 (2.76)	
6	236 (8.5)	46 (5.02)	73 (8.00)	115 (12.2)	
Outcome according to mRS					**< 0.001**
Good	1828 (65.9)	675 (73.7)	603 (66.1)	549 (58.3)	
Poor	946 (34.1)	241 (26.3)	309 (33.9)	393 (41.7)	

Continuous and categorical variables are expressed as median (interquartile range) and frequencies (percentages), respectively. Differences between groups have been tested using χ^2^ and U Mann-Whitney tests, as appropriate

ACR: acute-to-chronic glycaemic ratio; BMI: body mass index; HDL: high-density lipoprotein; LDL: low-density lipoprotein; mRS: Modified Rankin score; NIHSS: National Institutes of Health Stroke Scale; rtPA: recombinant tissue plasminogen activator; TOAST: Trial of ORG 10172 in acute stroke treatment

### Baseline characteristics according to ACR tertiles

We observed that age and presence of atrial fibrillation were positively correlated with ACR (all p-values < 0.05). On the other hand, we found a U-shapped association between ACR and history of diabetes and dyslipidemia, such that subjects in the first and third ACR tertiles were those with the highest prevalence of these risk factors.

Regarding stroke outcomes, baseline NIHSS, mRS at 3 months and indication of endovascular treatment were all positively correlated with ACR tertiles (all *p*-values < 0.05), observing no U-shapped relationship in this case. Besides, we compared log-ACR between patients with a good and poor prognosis observing a higher ACR in the latter group (*p* < 0.001), such that patients with a poor outcome showed on average a 1.07 (0.92–1.30) ACR value, while those with a good outcome presented a mean 0.99 (0.86–1.17) ACR value. The cohen’s d value of these differences was 0.29, suggesting a medium effect size in the univariate analyses (Additional Fig. 1). When we checked whether this relationship was influenced by the presence of diabetes, we observed no sign that diabetes moderated the relationship between stroke outcome and ACR, finding a non-significant interaction term (β = 0.00, 95% CI=-0.03 to 0.05, *p-*value = 0.686; Additional Fig. 2).

### Baseline characteristics according to stroke outcome

A univariate analysis was realized to assess the factors related to stroke outcomes. Previous mRS, age, baseline NIHSS, presence of cardiovascular risk factors and acute treatment were significantly associated to a poor prognosis. Moreover, patients with a poor prognosis had a higher baseline glucose and ACR (Additional Table 11).

### Multivariate analysis of continuous ACR and stroke outcome

Age, presence of diabetes, previous mRS, initial NIHSS and need of endovascular treatment were all associated with poor prognosis (Table 2, all p-values < 0.05). Regarding log-ACR, we observed that for each 0.1 increase in ACR, there was a 7% increase in the risk of presenting a poor outcome. When we entered the interaction term between log- ACR and diabetes, we observed no significant moderation (OR = 0.99, 95% CI = 0.93–1.06, p-value = 0.822). Similar results were observed when log-ACR was exchanged in the model for tertile-split ACR (Additional Table 2).

**Table 2:** Multivariate analysis of continuous ACR and stroke outcome (Rankin score 0–2 vs. 3–6)

	OR (95% CI)	*p* value
Male sex	0.84 (0.69–1.02)	0.084
Age	1.04 (1.03–1.05)	**< 0.001**
Diabetes	1.55 (1.27–1.90)	**< 0.001**
*Previous mRS*		
mRS-0	REF	REF
mRS-1	1.42 (1.08–1.86)	**0.011**
mRS-2	3.38 (2.57–4.45)	**< 0.001**
Baseline NIHSS	1.19 (1.16–1.21)	**< 0.001**
*Treatment*		
No treatment	REF	REF
Endovascular treatment	0.68 (0.42–1.09)	0.109
Endovascular + rTPA	0.50 (0.38–0.65)	**< 0.001**
ACR	1.07 (1.04–1.11)	**< 0.001**

Values represent Odds Ratios (OR), 95% confidence intervals and *p*-values

ACR: acute-to-chronic glycaemic ratio; NIHSS: National Institutes of Health Stroke Scale; rtPA: recombinant tissue plasminogen activator

### Multivariate analysis of ACR and stroke mortality

Regarding stroke mortality, we observed the same set of covariables associated with poor prognosis at 3 months. Log-ACR predicted a higher risk of stroke-related mortality, such that for each 0.1 increase in ACR we found a 10% increase in the odds of dying within the 3 months after stroke onset (Table 3). As we observed for stroke outcome, not significant interaction between log-ACR and diabetes was found (OR = 0.95, 95%CI = 0.87–1.04, p-value = 0.290). When ACR was split into tertiles, subjects in the third tertile presented 1.88-fold increased odds of mortality as compared to individuals in the first tertile (95% CI = 1.26–2.83, p-value = 0.002) (Additional Table 3).

**Table 3:** Multivariate analysis of continuous ACR and stroke mortality

	OR (95% CI)	*p* value
Male sex	1.13 (0.81–1.56)	0.472
Age	1.06 (1.04–1.08)	**< 0.001**
Diabetes	1.34 (0.97–1.84)	0.073
Previous mRS		
mRS-0	REF	**REF**
mRS-1	1.48 (0.98–2.21)	**0.006**
mRS-2	1.59 (1.06–2.36)	**0.024**
Baseline NIHSS	1.17 (1.15–1.20)	**< 0.001**
Treatment		
No treatment	REF	REF
Endovascular treatment	0.73 (0.41–1.28)	0.281
Endovascular + rTPA	0.56 (0.38–0.83)	**0.004**
ACR	1.10 (1.05–1.15)	**< 0.001**

Values represent Odds Ratios (OR), 95% confidence intervals and *p*-values

ACR: acute-to-chronic glycaemic ratio; NIHSS: National Institutes of Health Stroke Scale; rtPA: recombinant tissue plasminogen activator

### Predictive model of stroke prognosis

After splitting the sample into train (75%) and test (25%) sets, we used data from the training to build a baseline clinical model. This included the following variables: age, gender, diabetes, dyslipidemia, revascularization treatment, history mRS and baseline NIHSS. These variables minimize the AIC after testing all the possible combinations of variables (see the methods section). When we analyzed the performance of this model in the test set, we found an AUC of 0.781 (95% CI = 0.741–0.821).

**Fig. 1:**
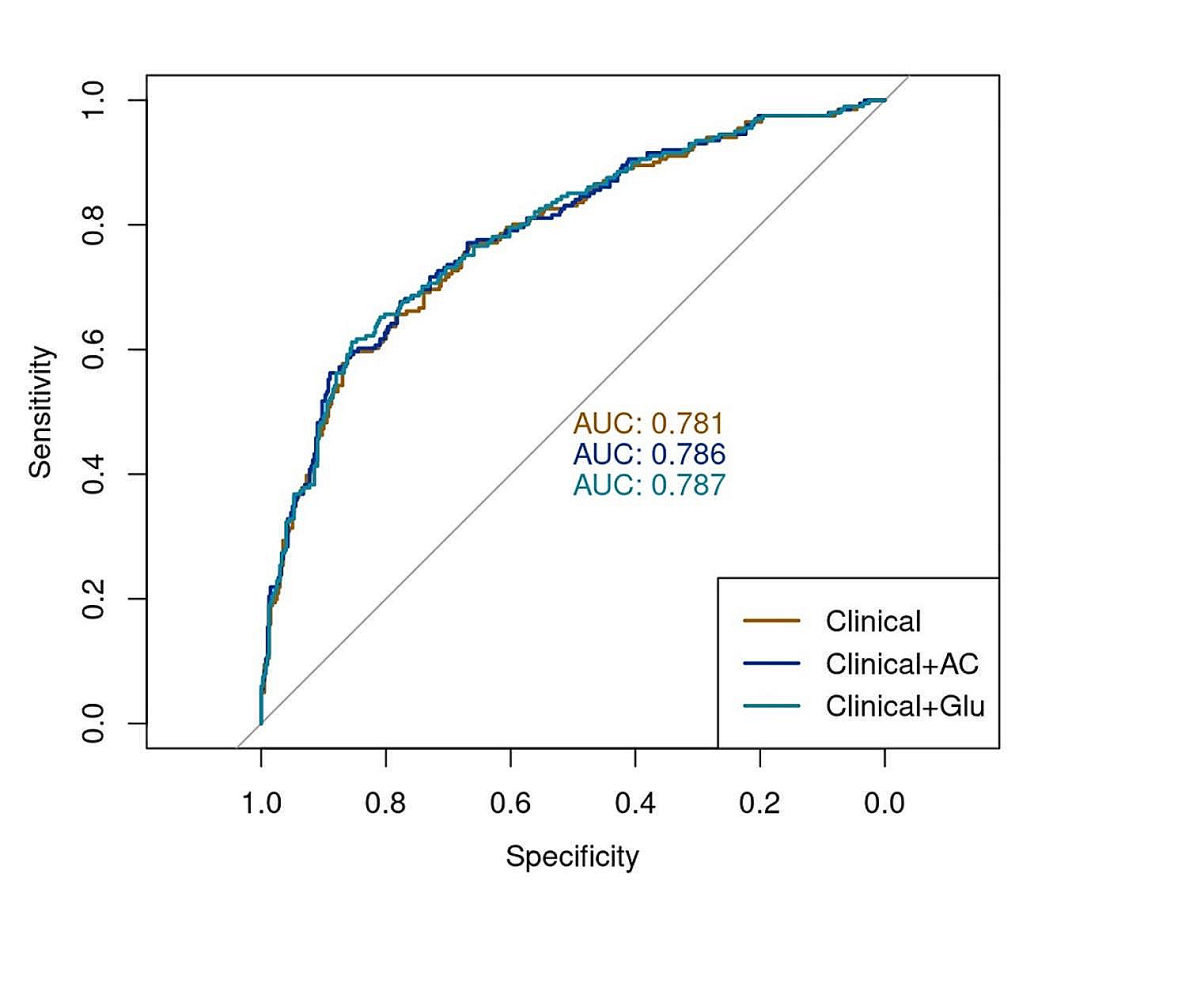
ROC curve comparing classical clinical variables vs. adding ACR o baseline glucose in the prediction of poor stroke outcome and mortality. **Model**: Rankin score > 1, age, gender, diabetes, dyslipidemia, revascularization treatment, history Rankin, baseline NIHSS

We subsequently built two additional models in the training set: one incorporating initial glycemia into the clinical model, and the other incorporating continuous ACR. The performance of these three models in terms of the AUC is depicted in Fig. 1, revealing no discernible differences among them. This finding was further validated through DeLong’s test for the models including initial glycemia (Z= −1.83, p-value = 0.067) and ACR (Z= −1.69, p-value = 0.091). Furthermore, in AdditionalFig. 3, we compare the performance of these models using various metrics (accuracy, sensitivity, specificity, F1-score), reaffirming their equivalence in terms of overall performance. Specifically, all models presented a relatively higfh specificity (Clinical = 0.89, Clinical + Gly = 0.89, Clinical + AC = 0.89), while a low sensitivity (Clinical = 0.51, Clinical + Gly = 0.52, Clinical + AC = 0.53). Finally, the F1-score measured the balance between sensitivity and specificity, confirming also that models were equivalent in terms of general performance (Clinical = 0.59, Clinical + Gly = 0.60, Clinical + AC = 0.61).

## Discussion

In the present study, we observed a positive and independent association between ACR and poor prognosis at 3 months, this association not influenced by the presence of diabetes. When categorizing into 3 ACR tertiles, we observed that being in the 3rd ACR tertile was also associated with a poor prognosis.

Acute hyperglycemia is, as previously mentioned, a frequent condition in neurological patients both with and without diabetes, and is related to a worse clinical prognosis [14, 15]. In this respect, the acute dysregulation in glucose metabolism can be evaluated using diverse parameters, including fasting glucose, admission random glucose, maximum glucose during the acute stage or HbA1c [16, 17]. Following this line, Sung et al. [18], focused on neurological patients and compared admission glucose, FBG and HbA1c in predicting neurological outcomes in subjects with AIS diagnosis. They concluded that fasting glucose was an independent predictor of poor neurological outcomes and had greater predictive power than that of admission glucose and HbA1c, not differing between subjects with and without diabetes. On the contrary, Roquer et al. [19] in 2015 published a study based on similar population cohort as the present study and observed that baseline glucose correlated with stroke severity in nondiabetic and diabetic patients with good previous glucose control (HbA1c < 7%), but not in those with poor glucose control (HbA1c > 7%).

Recent indexes and ratios have been established to further evaluate stress hyperglycemia, thus taking into account both acute and chronic glucose control [9, 20]. These include the previously mentioned ACR, or others such as the stress hyperglycemia ratio (SHR) or FBG / HbA1c ratio [8]. It has been hypothesized that they could play a more relevant role as outcome-predictive factors than only glucose values or HbA1c separately.

Considering neurological patients, to the best of our knowledge, the present study is one of the first to evaluate ACR as an outcome predictor after AIS diagnosis, confirming its relationship with a poor outcome and mortality at 3-month follow-up. Moreover, baseline NIHSS was also one of the observed mortality predictive factors. In this same line, a recent study including a total of 335 subjects with diabetes and AIS diagnosis specifically focused on the correlation between ACR and illness severity [12]. Despite the limited number of patients included, interestingly, when stratifying patients according to admission NIHSS, no differences were found in fasting glucose, admission glucose or HbA1c levels, although ACR was significantly different, hence highlighting the possible superiority of ACR as an outcome predictive factor in comparison to the other glycemic variables for separate. Another recent publication also observed a correlation between increased ACR and poor outcomes. However, in this publication, only 18.1% percentage of the subjects included had a previous diabetes diagnosis [21], in comparison to almost 35% of subjects with previous diabetes diagnoses in the present study. Age was positively associated with a poor prognosis at 3 months while revascularization therapy was found to be a protective factor, in accordance with previous publications [18]. Moreover, it is worth highlighting that, in the present study, despite less than 40% of the included subjects having diabetes diagnosis before admission, the imbalance between acute and chronic glycemic control continued to be an outcome-predictive factor during follow-up. A very recent study [22], in this case evaluating the FBG/ HbA1c ratio and not ACR, observed this ratio to be associated with more severe AIS. Specifically, the glucose-to-HbA1c ratio was associated with functional outcomes in patients without diabetes but not in patients with diabetes. These results highlight the importance of stress hyperglycemia in subjects with AIS diagnosis, even in the absence of an alteration in glucose metabolism before hospital admission.

The present study also divided the included patients according to 3 different ACR tertiles. It was observed that being included in the 3rd ACR tertile (> 1.13), increased the risk of a poor outcome by 62% and mortality by 88%. Hence, these results emphasize the fact that presenting a glucose level at admission 13% higher than expected has a detrimental effect on AIS prognosis. Following this same line, several previous publications also observed an association between glucose / HbA1c ratios and clinical outcomes in both patients with and without diabetes diagnosis [16, 17, 23, 24] although most of them did not categorize into different tertiles.

Moreover, since those subjects included in the top tertile present an increased risk of poor prognosis, it seems plausible to think that this subgroup of patients could benefit from a more intensive insulin therapy. However, it must be considered that, as observed in previous publications focused on critical patients [25, 26], a more intensive glucose control can also be associated with an increased mortality risk, secondary to a higher hypoglycemia rate. Hence, if we want to normalize acute high glucose levels of the 3rd ACR tertile group without increasing the presence of hypoglycemia, a precise blood glucose monitoring system seems essential. In this regard, the use of continuous glucose monitoring (CGM), which can continuously and automatically provide instant blood glucose values together with pre-specified hyper and hypoglycemia alarms, could play an important role in glucose management in these patients, as has previously demonstrated in intensive-care unit (ICU) patients [27]. Thus, combining intensive insulin therapy with CGM to fight stress hyperglycemia in the 3rd ACR tertile group in clinical daily practice could presumably have a direct effect on the patient’s prognosis in the short-term after AIS diagnosis.

Finally, the present study went one-step further, analyzing the possible clinical relevance of ACR in this subgroup of neurological patients. In this respect, it must be acknowledged that, although several scores have been defined to evaluate global cardiovascular risk [28, 29], to date no risk score has been specifically defined for stroke patients. Owing to all this, we created a model which included classical risk factors for neurological patients such as age, gender, diabetes, dyslipidemia, revascularization treatment, history Rankin and baseline NIHSS. This classical model obtained an AUC of 0.781 in the ROC curve, without a significant increase in the AUC when baseline glucose or ACR were included in the model. Hence, although the results in the present study are encouraging as they observe a significant and independent association between ACR and poor prognosis in the short term after AIS, the clinical relevance of this ratio still needs to be further defined.

This study is not without limitations. The study was realized in a single center, and hence this may limit the extrapolation of the results. Moreover, a retrospective analysis was realized. The relative short period of follow-up must also be taken into account, together with the possible presence of other confounding factors that could have affected the final results. Finally, diabetes diagnosis was defined using different variables, including previous medical history or use of hypoglycemic medication, but laboratory results of glucose values or HbA1c to confirm diagnosis were not available for all the included subjects.

## Conclusions

ACR was positively associated with a poor prognosis and mortality 3 months after AIS diagnosis. Other factors identified as being associated with poor outcome at 3 month-follow up included age, presence of diabetes, previous mRS and stroke severity, while revascularization therapy demonstrated a protective effect.

Individuals in the third tertile of ACR showed a poorer prognosis and higher mortality rate. Finally, the addition of baseline glucose or ACR on a model with classical clinical factors did not provide additional predictive value regarding stroke prognosis.

Therefore, future studies and needed to further elucidate the specific role of ACR in clinical daily practice.

### Supplementary Information

Supplementary material 1: Differences levels of ACR between patients with good and poor prognosis and by presence of diabetes.

Supplementary material 2: Differences in ACR by stroke outcome.

Supplementary material 3: Performance of the different models used for the predication of poor stroke outcome and mortality.

Supplementary material 4: Tables.

## Data Availability

No datasets were generated or analysed during the current study.
