# Supplementary figures and images for: Acute-to-chronic glycemic ratio as an outcome predictor in ischemic stroke in patients with and without diabetes mellitus
Source: Cardiovasc Diabetol. 2024 Jun 18;23:206. doi: 10.1186/s12933-024-02260-9 (PMC11186093; doi:10.1186/s12933-024-02260-9)

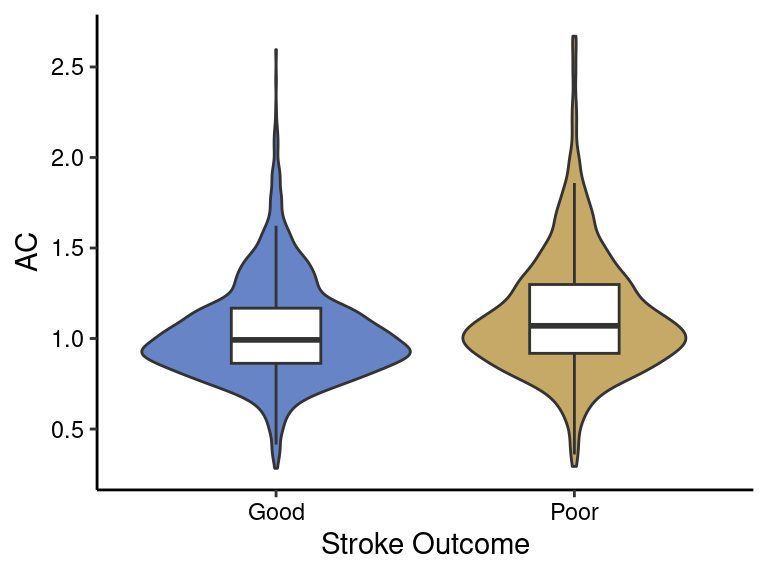

Supplement: Supplementary file 1 — Supplementary material 1: Differences levels of ACR between patients with good and poor prognosis and by presence of diabetes. [file 12933_2024_2260_MOESM2_ESM.docx]

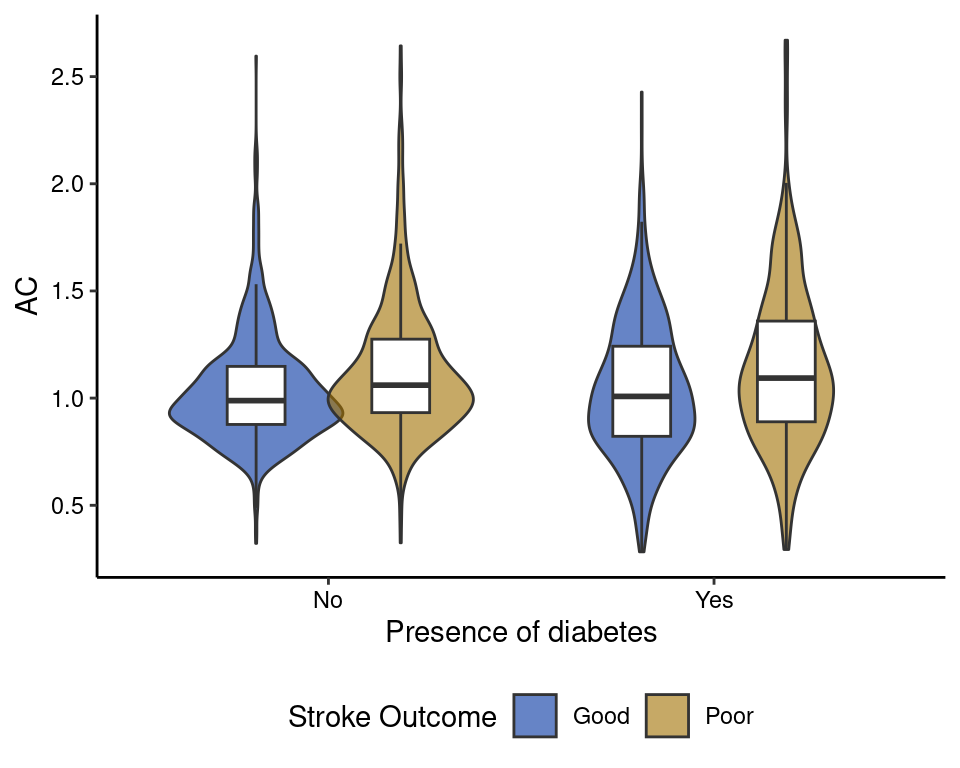

Supplement: Supplementary file 2 — Supplementary material 2: Differences in ACR by stroke outcome. [file 12933_2024_2260_MOESM1_ESM.docx]

**
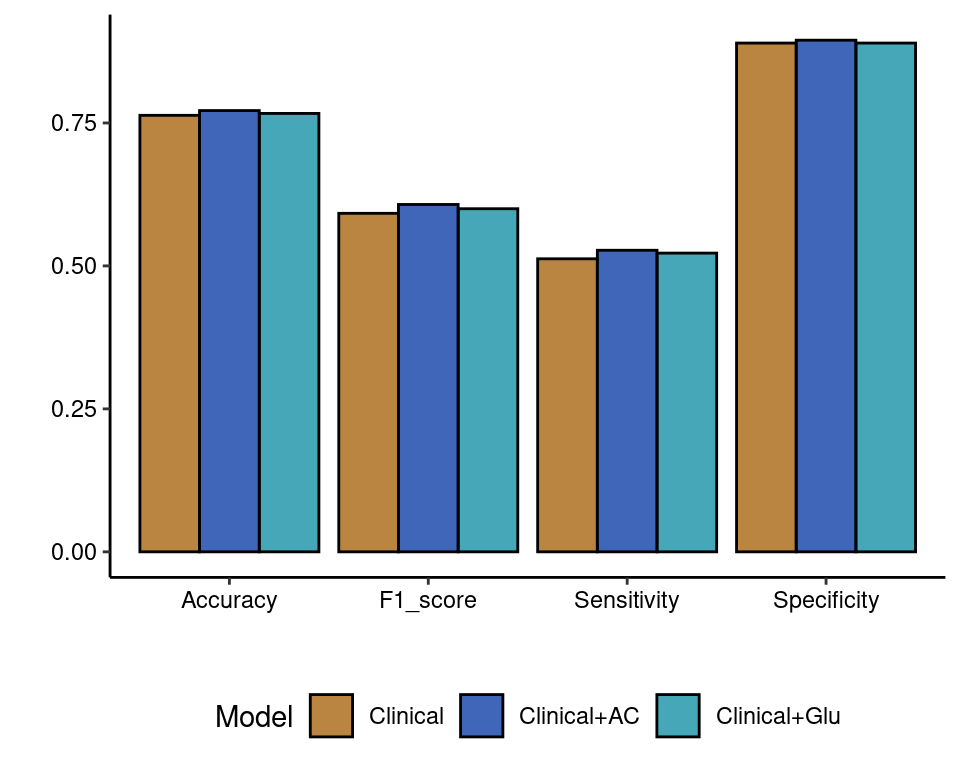
**

Supplement: Supplementary file 3 — Supplementary material 3: Performance of the different models used for the predication of poor stroke outcome and mortality. [file 12933_2024_2260_MOESM3_ESM.docx]
