# Supplementary material for: Acute-to-chronic glycemic ratio as an outcome predictor in ischemic stroke in patients with and without diabetes mellitus
Source: Cardiovasc Diabetol. 2024 Jun 18;23:206. doi: 10.1186/s12933-024-02260-9 (PMC11186093; doi:10.1186/s12933-024-02260-9)
Supplement: Supplementary file 4 — Supplementary material 4: Tables. [file 12933_2024_2260_MOESM4_ESM.docx]

**Supplementary Table 1.** Univariate analysis of factors associated to stroke outcomes and mortality

| **Variable** | **Good prognosis** | **Poor prognosis** | **p value** |
| --- | --- | --- | --- |
| N | 1828 | 946 |  |
| Gender | | | **<0.001** |
| Male | 1107 (60.6) | 440 (46.6) |  |
| Female | 720 (39.4) | 505 (53.4) |  |
| Age, years | 71.0 (60.0-80.0) | 79.0 (71.0-85.0) | **<0.001** |
| Hypertension | 1312 (71.8) | 750 (79.4) | **<0.001** |
| Dyslipidemia | 953 (52.4) | 454 (48.2) | **0.042** |
| Diabetes | 582 (31.8) | 377 (39.9) | **<0.001** |
| Coronary heart disease | 252 (13.8) | 147 (15.7) | 0.219 |
| Atrial fibrillation | 445 (24.3) | 399 (42.2) | **<0.001** |
| BMI (kg/m2) | 26.8 (24.2-29.9) | 27.0 (23.9-29.6) | 0.536 |
| Systolic blood pressure (mmHg) | 153 (137-175) | 154 (136-175) | 0.824 |
| Diastolic blood pressure (mmHg) | 81.0 (71.0-91.0) | 80.0 (70.0-90.0) | **0.009** |
| rtPA treatment | 338 (18.8) | 193 (20.8) | 0.223 |
| Endovascular treatment | 394 (21.8) | 269 (28.8) | **<0.001** |
| Baseline NIHSS | 3.00 (2.00-6.00) | 9.00 (4.00-17.0) | **<0.001** |
| TOAST | | | **<0.001** |
| Atherotrombotic | 288 (15.8) | 137 (14.5) |  |
| Cardioembolic | 493 (27.0) | 394 (41.6) |  |
| Lacunar | 512 (28.0) | 119 (12.6) |  |
| Undetermined | 482 (26.4) | 256 (27.1) |  |
| Unusual | 53 (2.90) | 40 (4.23) |  |
| Baseline mRS | | | **<0.001** |
| 0 | 1461 (79.9) | 551 (58.2) |  |
| 1 | 228 (12.5) | 164 (17.3) |  |
| 2 | 139 (7.60) | 231 (24.4) |  |
| Glucose (mg/dL) | 117 (100-150) | 131 (109-172) | **<0.001** |
| HbA1c (%) | 5.80 (5.40-6.50) | 5.80 (5.40-6.90) | **0.007** |
| HbA1c (mmol/mol) | 40 (34-48) | 40 (34-52) | **0.007** |
| ACR | 0.99 (0.86-1.17) | 1.07 (0.92-1.30) | **<0.001** |
| ACR tertiles | | | **<0.001** |
| Tertile 1 (0.28-0.92) | 675 (36.9) | 241 (25.6) |  |
| Tertile 2 (0.92-1.13) | 603 (33.0) | 309 (32.8) |  |
| Tertile 3 (> 1.13) | 549 (30.0) | 393 (41.7) |  |

SI conversion factors: To convert glucose to mmol/L, multiply values by 0.0555

Continuous and categorical variables are expressed as median (interquartile range) and frequencies (percentages), respectively.

ACR: acute-to-chronic glycaemic ratio; BMI: body mass index; HDL: high-density lipoprotein; LDL: low-density lipoprotein; mRS: Modified Rankin score; NIHSS: National Institutes of Health Stroke Scale; rtPA: recombinant tissue plasminogen activator; TOAST: Trial of ORG 10172 in acute stroke treatment

**Supplementary Table 2.** Multivariate analysis of ACR tertiles and stroke outcome (Rankin score 0-2 vs 3-6).

|  | **OR (95% CI)** | **p value** |
| --- | --- | --- |
| Male sex | 0.84 (0.69-1.02) | 0.085 |
| Age | 1.04 (1.03-1.05) | **< 0.001** |
| Diabetes | 1.59 (1.30-1.94) | **< 0.001** |
| Previous mRS |  |  |
| mRS-0 | REF | REF |
| mRS-1 | 1.41 (1.08-1.85) | **0.013** |
| mRS-2 | 3.43 (2.61-4.51) | **< 0.001** |
| Baseline NIHSS | 1.19 (1.16-1.21) | **< 0.001** |
| Treatment |  |  |
| No treatment | REF | REF |
| Endovascular treatment | 0.68 (0.42-1.09) | 0.109 |
| Endovascular + rTPA | 0.50 (0.38-0.65) | **< 0.001** |
| ACR Tertile 2 (0.921-1.13) | 1.45 (1.14-1.85) | **0.003** |
| ACR Tertile 3 (1.13-2.67) | 1.62 (1.28-2.06) | **< 0.001** |

Values represent Odds Ratios (OR), 95% confidence intervals and *p*-values.

ACR: acute-to-chronic glycaemic ratio; NIHSS: National Institutes of Health Stroke Scale; rtPA: recombinant tissue plasminogen activator

**Supplementary Table 3.** Multivariate analysis of ACR tertiles & stroke mortality

|  | **OR (95% CI)** | **p value** |
| --- | --- | --- |
| Male sex | 1.11 (0.80-1.53) | 0.534 |
| Age | 1.06 (1.04-1.08) | **< 0.001** |
| Diabetes | 1.40 (1.01-1.92) | **0.040** |
| Previous mRS |  |  |
| mRS-0 | REF | REF |
| mRS-1 | 1.48 (0.98-2.21) | 0.061 |
| mRS-2 | 1.64 (1.1-2.44) | **0.015** |
| Baseline NIHSS | 1.18 (1.15-1.21) | **< 0.001** |
| Treatment |  |  |
| No treatment | REF | REF |
| Endovascular treatment | 0.74 (0.41-1.29) | 0.305 |
| Endovascular+rTPA | 0.55 (0.37-0.82) | **0.003** |
| ACR Tertile 2 (0.921-1.13) | 1.51 (0.98-2.34) | 0.064 |
| ACR Tertile 3 (1.13-2.67) | 1.88 (1.26-2.83) | **0.002** |

Values represent Odds Ratios (OR), 95% confidence intervals and *p*-values.

ACR: acute-to-chronic glycaemic ratio; NIHSS: National Institutes of Health Stroke Scale; rtPA: recombinant tissue plasminogen activator
